# A successful case of varix of the left gastroepiploic vein preoperatively diagnosed by 3D-CT angiography and resected by laparoscopy: A case report

**DOI:** 10.1097/MD.0000000000025347

**Published:** 2021-04-23

**Authors:** Kentaro Matsuo, Sang-Woong Lee, Ryo Tanaka, Yoshiro Imai, Kotaro Honda, Kazuhiro Yamamoto, Kazuhisa Uchiyama

**Affiliations:** a Department of General and Gastroenterological Surgery; b Department of Diagnostic Radiology, Osaka Medical College, 2-7, Daigaku-machi, Takatsuki, Osaka, Japan.

**Keywords:** 3D-CT angiography, successful case, varix of the left gastroepiploic vein

## Abstract

**Introduction:**

Gastric varices can be present in up to 20% of patients with portal hypertension. However, a varix of the left gastroepiploic vein (LGV) is extremely rare. Surgery is required if bleeding occurs; thus, precise diagnosis is crucial. We present a successful case of preoperative diagnosis intraabdominal varix of the LGV using three-dimensional-computed tomography angiography (3D-CTA) followed by laparoscopic resection. This is the first report of a case with variant LGV. Our study demonstrates the efficacies of 3D-CTA and laparoscopic surgery for the diagnosis and safe resection of the intraabdominal varix, respectively.

**Patient concerns:**

A 74-year-old woman was referred to our department with a tumor in the abdominal cavity. On physical examination, no lumps were palpable in the upper abdomen.

**Diagnosis:**

The enhanced CT was revealed that the tumor was not enhanced in the early phase, but in the equilibrium phase. Moreover, 3D-CTA clearly revealed that the tumor was being supplied by the LGV. Thus, it was diagnosed as a variant of the LGV.

**Interventions:**

Surgical resection was performed laparoscopically as per the guidance of preoperative 3D-CTA findings. During surgery, a dark tumor was found along the gastroepiploic vessels, supplied by the LGV. The tumor was resected safely based on the preoperative information.

**Outcomes:**

Histopathological examination of the tumor showed accumulation of various vessels, but no malignant cells. Therefore, we made a final diagnosis of the tumor as an LGV varix. For follow-up, an annual CT examination was performed and after 3 years postoperation, no recurrence was observed.

**Conclusions:**

In the present case, we have achieved a successful preoperative diagnosis using 3D-CTA, and resection was safely accomplished using laparoscopy guided by preoperative anatomical information. This is the first report of an LGV variant. Appropriate management is crucial because bleeding is a catastrophic event. Therefore, imaging procedures such as 3D-CTA for diagnosis, followed by safe resection by laparoscopic surgery, are effective tools for the treatment of epiploic vein varices.

## 1 Introduction

Gastric varices may be present in up to 20% of patients with portal hypertension and have a 65% risk of bleeding due to the high intravariceal pressure.^[1,2]^ Although the mortality rate of bleeding varices is still high, treatment is becoming more effective due to the development of endoscopic devices and techniques.^[3]^ However, there are currently no reports of varices of the left gastroepiploic vein (LGV), and it is not yet understood how these varices may occur and what they interact with. Moreover, surgical treatment is required once the varix is ruptured; thus, it is important to diagnose it appropriately and resect it safely before rupture occurs.

Here, we report a diagnosis of a varix of the LGV using preoperative 3D-CTA, followed by safe resection using laparoscopic surgery.

## 2 Case presentation

An asymptomatic 74-year-old woman was referred to our department with a tumor in the abdominal cavity. The tumor was more than 20 mm in size and was initially suspected to be an aneurysm of the epiploic artery, as suggested by enhanced CT findings. On physical examination, no lumps were palpable in the upper abdomen, while laboratory investigations showed slight anemia (Hb value: 10.5 g/dl), no liver dysfunction, and normal levels of tumor markers (CEA, 1.3 ng/ml; CA-19-9, 4.2 U/ml).

**Figure 1:**
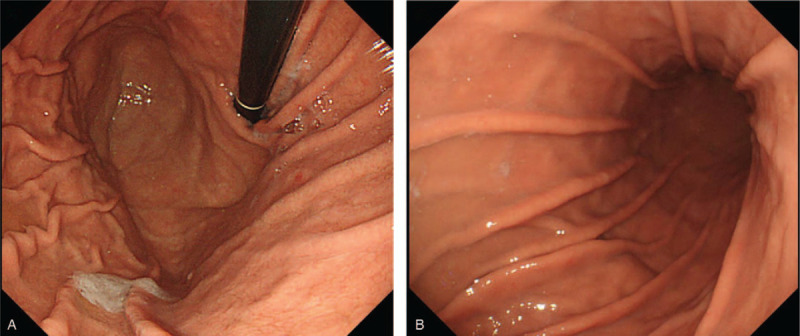
Upper gastrointestinal endoscopic images. (A) No gastric varices were observed at the cardia and fornix. (B) No gastric varices were observed at the body and antrum.

**Figure 2:**
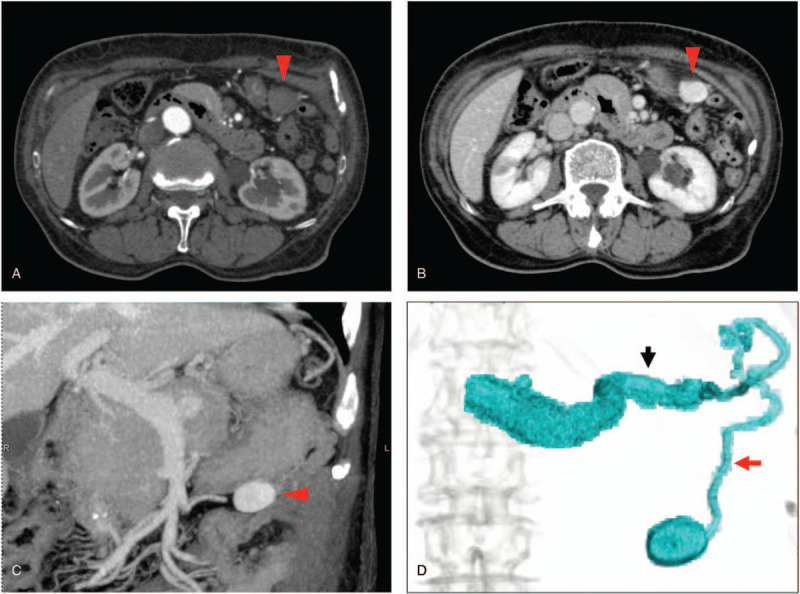
Representative preoperative images. (A) An axial contrast-enhanced CT image in the early phase demonstrating that the tumor of the abdominal cavity was not enhanced heterogeneously (red arrowhead). (B) The same area presented in the late phase, showing enhancement (red arrowhead). (C) A coronal enhanced-CT image in the late phase. These images demonstrate that the enhanced tumor was situated at the greater curvature (red arrowhead). (D) 3D-CTA. These images demonstrate that the tumor was supplied from the LGV (red arrow) and the splenic vein (black arrow).

Upper gastrointestinal endoscopy could not detect any tumors or varices in the esophagus or stomach (Fig. 1A and B). An enhanced CT demonstrated that the tumor was not enhanced in the early phase (Fig. 2A), but in the equilibrium phase (Fig. 2B and C). Despite these findings, an upper gastrointestinal endoscopy did not detect tumor or varices in the esophagus or stomach (Fig. 1A and \B). However, 3D-CTA clearly revealed that the tumor was being supplied by the LGV (Fig. 2D). Consequently, the tumor was diagnosed as a variant of the LGV and surgical resection under the guidance of the preoperative 3D-CTA findings was planned.

**Figure 3:**
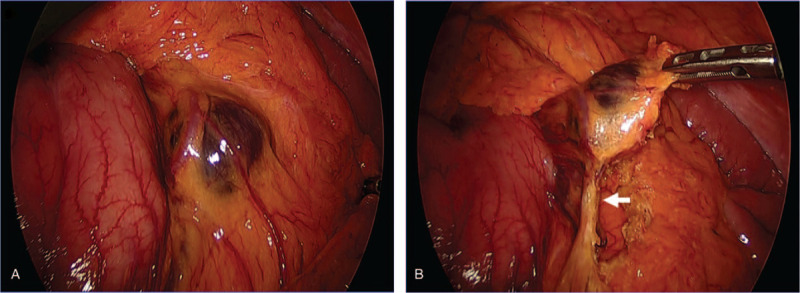
Highlights of the intraoperative images. (A) The tumor was positioned along the greater curvature as shown in the enhanced CT. (B) The tumor originated from the LGV. The white arrow indicates the LGV following toward the tumor.

During surgery, a dark tumor was found along the gastroepiploic vessels. The tumor had mobility without any adhesion to or invasion of adjacent organs (Fig. 3A). The LGV was identified and found to be supplying the tumor (Fig. 3B). The tumor was resected safely based on preoperative information.

**Figure 4:**
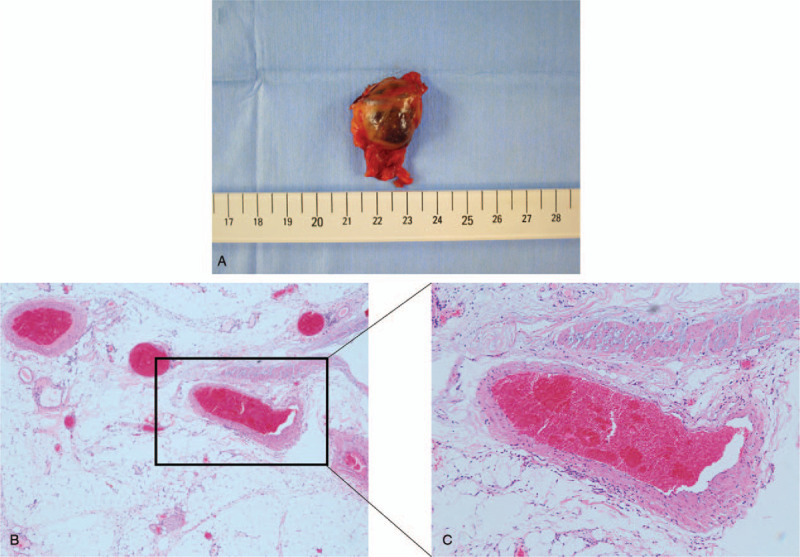
Highlights of the postoperative and microscopic images. (A) The tumor measured 20 mm in size and revealed a hemorrhagic solid mass. (B and C) Hematoxylin-eosin staining showing the accumulation of vessels of various sizes. No malignancy was identified. (B: image at 20× magnification C: image at 40× magnification).

The tumor was 20 mm in size and the surface was macroscopically smooth and soft (Fig. 4A). Histopathological examination showed an accumulation of various vessels cells but no malignant cells (Fig. 4B and C); therefore, we finally made a diagnosis of a LGV varix.

In this case, we accurately diagnosed a LGV varix using preoperative 3D-CTA, and subsequently performed safety resection by laparoscopic surgery. For follow-up, an annual CT examination was performed and after 3 years postoperation, there was no sign of any recurrence.

## 3 Discussion and conclusions

Although their etiology is unclear, gastric varices mainly occur due to portal hypertension and abnormal shunts.^[2,4]^ Once bleeding occurs, it is considered an emergency event with a high mortality rate. Therefore, preventing any hemorrhage is crucial to avoid the need for endoscopic or surgical treatment.^[3]^ The diagnosis and handling of gastroepiploic vein varices are poorly understood, as they are extremely rare entities.

Preoperative 3D-CTA is a useful tool to diagnose and detect the pattern of blood supply to a tumor. We previously reported that we could diagnose the giant gastrointestinal stromal tumors (GISTs) nourished by the right epiploic artery by using 3D-CTA.^[5]^ Indeed, in the present case, once we suspected an abdominal tumor, we investigated it initially using abdominal CT.^[6]^ Then, we attempted to diagnose it more specifically by using 3D-CTA. As shown in Figure 2, the tumor was not enhanced in the early phase but was prominent in the late phase, and it was clear that the tumor was supplied by the LGV. If this tumor would have been a malignant or submucosal tumor, it should have been enhanced in the early phase and been supplied by the artery. Thus, we were able to rule out the possibility of its malignancy preoperatively and to make a precise diagnosis of the tumor as a varix. We performed a laparoscopic resection based on preoperative anatomical information.

Laparoscopic examination is a useful technique because it is possible to observe the tumor directly and treat it with minimal invasion, regardless of the difficulty of preoperative diagnosis.^[7,8]^ Moreover, the varix must be removed to prevent critical events and surgery. In this case, laparoscopic surgery was performed, and it was possible to determine the location and condition of the tumor without the need for a large incision. The patient had a good postoperative course; thus, laparoscopic treatment is an additional option in the case of an abdominal tumor that is difficult to diagnose preoperatively.

In conclusion, this is the first report on a variant of LGV. Effective management of LGV varices is crucial because bleeding is considered a critical event. Therefore, making full use of an imaging procedure such as 3D-CTA and safety resection by laparoscopic surgery are effective tools for epiploic vein varix.

## Acknowledgments

We would like to thank Editage (www.editage.com) for English language editing.

## Author contributions

KM, RT, YI, KH, and SL provided care to the patient, including the surgery. KY created the 3D-CTA. KM and SL designed and drafted the manuscript. KU reviewed and revised the manuscript. All authors have read and approved the final manuscript.

**Data curation:** Kentaro Matsuo, Sang-Woong Lee, Ryo Tanaka, Yoshiro Imai, Kotaro Honda, Kazuhiro Yamamoto.

**Investigation:** Kazuhiro Yamamoto.

**Supervision:** Sang-Woong Lee, Kazuhisa Uchiyama.

**Writing – original draft:** Kentaro Matsuo.

**Writing – review & editing:** Sang-Woong Lee, Kazuhisa Uchiyama.
